# Impacts of neoadjuvant therapy on the number of dissected lymph nodes in esophagogastric junction cancer patients

**DOI:** 10.1186/s12876-023-02705-7

**Published:** 2023-03-09

**Authors:** Qi Wang, Jin-tong Ge, Hua Wu, Sheng Zhong, Qing-quan Wu

**Affiliations:** Department of Thoracic Surgery, The Affiliated Huaian No.1 People’s Hospital of Nanjing Medical University, Huaian, 223300 Jiangsu China

**Keywords:** Esophagogastric junction cancer, Radiotherapy, Chemoradiotherapy, Chemotherapy, Lymph node

## Abstract

**Background:**

Neoadjuvant therapy favors the prognosis of various cancers, including esophagogastric junction cancer (EGC). However, the impacts of neoadjuvant therapy on the number of dissected lymph nodes (LNs) have not yet been evaluated in EGC.

**Methods:**

We selected EGC patients from the Surveillance, Epidemiology, and End Results (SEER) database (2006–2017). The optimal number of resected LNs was determined using X-tile software. Overall survival (OS) curves were plotted with the Kaplan–Meier method. Prognostic factors were evaluated using univariate and multivariate COX regression analyses.

**Results:**

Neoadjuvant radiotherapy significantly decreased the mean number of LN examination compared to the mean number of patients without neoadjuvant therapy (12.2 vs. 17.5, *P* = 0.003). The mean LN number of patients with neoadjuvant chemoradiotherapy was 16.3, which was also statistically lower than 17.5 (*P* = 0.001). In contrast, neoadjuvant chemotherapy caused a significant increase in the number of dissected LNs (21.0, *P* < 0.001). For patients with neoadjuvant chemotherapy, the optimal cutoff value was 19. Patients with > 19 LNs had a better prognosis than those with 1–19 LNs (*P* < 0.05). For patients with neoadjuvant chemoradiotherapy, the optimal cutoff value was 9. Patients with  > 9 LNs had a better prognosis than those with 1–9 LNs (*P* < 0.05).

**Conclusions:**

Neoadjuvant radiotherapy and chemoradiotherapy decreased the number of dissected LNs, while neoadjuvant chemotherapy increased it in EGC patients. Hence, at least 10 LNs should be dissected for neoadjuvant chemoradiotherapy and 20 for neoadjuvant chemotherapy, which could be applied in clinical practice.

## Introduction

Esophagogastric junction cancer (EGC) invades the esophagogastric junction and involves the anatomic border between the proximal stomach and esophagus, with an increasing incidence worldwide [1, 2]. The number and location of metastatic lymph nodes (LNs) are considered independent prognostic predictors in EGC patients [3–5]. However, the extent of LN dissection remains controversial. Wang et al. [6] showed that the dissection of parapyloric LN (No. 5 and 6 LNs, PLN) brought a survival benefit to Siewert type II/III EGC patients. Jun Peng et al. [7] recommended that at least 12 LNs should be examined to ensure accurate staging for Siewert type II EGC patients. In contrast, no significant survival differences were observed between patients with loco LN dissection alone and those with extended lymphadenectomy (including station 8/11) [8]. Different ethnicities might be the cause of these differences.

Due to strong invasive properties, a large fraction of EGC patients is detected at a locally advanced stage, and neoadjuvant therapy, including radiotherapy and chemotherapy, is required for most of them [9–11]. Accumulating evidence has shown that neoadjuvant therapy can effectively improve the clinical outcomes of EGC patients such as large clinical trials CheckMate 577 [12], PRODIGY [13], and CROSS [14]. Combined neoadjuvant immunotherapy and chemoradiotherapy have also demonstrated favorable efficacy and safety [15]. However, whether neoadjuvant therapy affects the number of dissected LNs remains unclear. A single-center, retrospective study has shown that neoadjuvant therapy decreased the number of dissected LNs in esophageal cancer patients [16]. Nevertheless, another study based on the Surveillance, Epidemiology, and End Results (SEER) database argued that there was no significant difference in the number of dissected LNs between esophageal cancer patients with preoperative radiotherapy and those without [17]. These inconsistent results imply that more studies are needed. Besides, these studies mainly focused on esophageal cancer rather than EGC. The impact of neoadjuvant therapy on the number of dissected LNs is yet to be fully evaluated in EGC patients. Therefore, in the present study, we explored whether neoadjuvant therapy impacted the number of LN examination and evaluated the optimal number of LN examination using the SEER database.

## Methods

### Patients

We retrieved patients from the SEER database (2006–2017). Patients between 2004 and 2005 were not included in the study because of the missing chemotherapy data in the SEER database. The inclusion criteria were as follows: (1) positive histology; (2) underwent radical surgery; (3) definite tumor stage (T-category) and nodal stage (N-category), according to the 8th edition of the American Joint Committee on Cancer (AJCC) criteria; (4) first primary tumor; (5) at least one regional lymph node dissection based on pathologic evidence; (6) treatment with/without preoperative radiotherapy and/or chemotherapy; and (7) no distant metastasis. The exclusion criteria were: (1) under 18 years old; (2) unavailable follow-up data; (3) unknown cause of death. We retrieved baseline characteristics, including the year of diagnosis, race, age, sex, histology, grade, marital status, T-category and N-category. The data from all subjects from the SEER database was obtained legally.

### Statistical analysis

Differences between patients with neoadjuvant therapy and those without were analyzed by χ^2^ and t-tests. Univariate and multivariate analysis were performed with a Cox proportional hazards regression model to explore prognostic factors for EGC patients with neoadjuvant therapy. The optimal number of LN examination was explored using X-tile software (Yale University, Version 3.6.1). The overall survival (OS) was analyzed with a Kaplan–Meier analysis. Data were analyzed with PASW Statistics 18. A two-sided *p*-value < 0.05 was considered to indicate a significant difference.

## Results

### Baseline characteristics

**Fig. 1:**
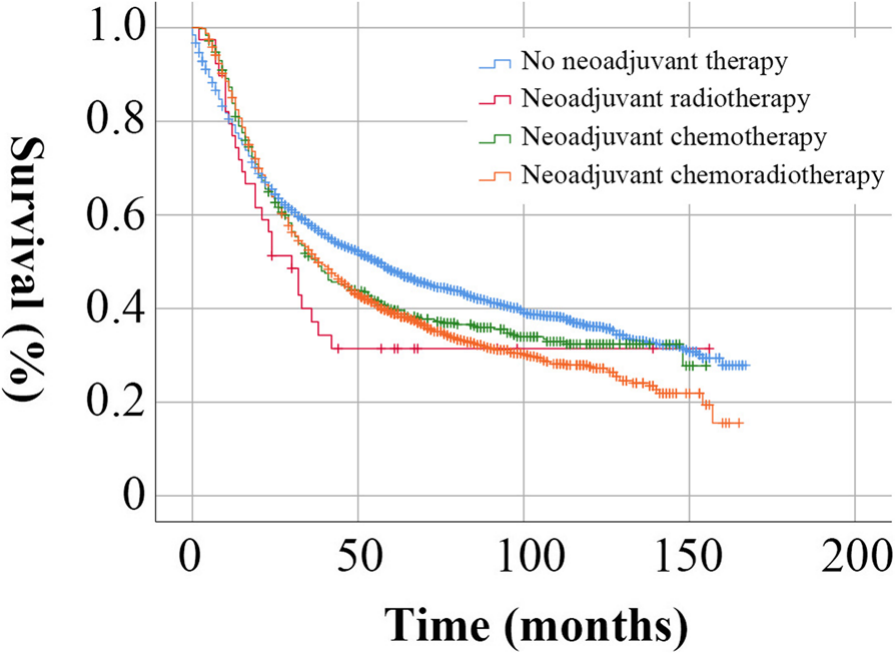
Survival curves of the entire cohort according to the treatment modality

We enrolled 4028 EGC patients, including 2020 without neoadjuvant therapy, 498 with neoadjuvant chemotherapy, 39 with neoadjuvant radiotherapy, and 1471 with neoadjuvant chemoradiotherapy. The baseline characteristics are summarized in Table 1. The median age of the entire cohort was 64 years (range: 20–94 years), and more patients received neoadjuvant therapy during 2012–2017 than in 2006–2011. Males and adenocarcinoma accounted for the majority in the cohort. The highest proportion of T-category was T1 and T2 among patients without neoadjuvant therapy, while T2 and T3 were predominant among patients with neoadjuvant therapy. The highest proportion of N-category was N0 among patients without neoadjuvant therapy, while N1 was predominant among patients with neoadjuvant therapy. These results indicated that patients with higher tumor stages tended to receive neoadjuvant therapy. The survival analysis showed that patients with neoadjuvant therapy had a worse prognosis than those without neoadjuvant therapy (Fig. 1, *P* < 0.05).

**Table 1:** Baseline characteristics of EGC patients

**Characteristics**	**No neoadjuvant therapy**	**Neoadjuvant therapy**	**Neoadjuvant chemotherapy**	**Neoadjuvant radiotherapy**	**Neoadjuvant chemoradiotherapy**	***P*** **-value (No treatment vs. treatment)**
**N**	2020	2008	498	39	1471	
**Year of diagnosis**						< 0.001
2006–2011	1271 (62.9%)	905 (44.8%)	273 (54.8%)	18 (46.2%)	614 (41.7%)	
2012–2017	749 (37.1%)	1103 (55.2%)	225 (45.2%)	21 (53.8%)	857 (58.3%)	
**Race**						0.425
White	1726 (85.4%)	1799 (89.6%)	419 (84.1%)	33 (84.6%)	1347 (91.6%)	
Black	99 (4.9%)	77 (3.8%)	28 (5.6%)	4 (10.3%)	45 (3.0%)	
Others	195 (9.7%)	132 (6.6%)	51 (10.3%)	2 (5.1%)	79 (5.4%)	
**Marital status**						0.004
Married	1351 (66.9%)	1427 (71.1%)	357 (71.7%)	22 (56.4%)	1048 (71.2%)	
Others	669 (33.1%)	581 (28.9%)	141 (28.3%)	17 (43.6%)	423 (28.8%)	
**Age**						< 0.001
≤ 60	656 (32.5%)	849 (42.3%)	220 (44.2%)	17 (43.6%)	612 (41.6%)	
> 60	1364 (67.5%)	1159 (57.7%)	278 (55.8%)	22 (56.4%)	859 (58.4%)	
**Sex**						< 0.001
Male	1570 (77.7%)	1702 (84.8%)	414 (83.1%)	36 (92.3%)	1252 (85.1%)	
Female	450 (22.3%)	306 (15.2%)	84 (16.9%)	3 (7.7%)	219 (14.9%)	
**Histology**						< 0.001
Adenocarcinoma	1731 (85.7%)	1602 (79.8%)	330 (66.3%)	31 (79.5%)	1241 (84.4%)	
Others	289 (14.3%)	406 (20.2%)	168 (33.7%)	8 (20.5%)	230 (15.6%)	
**T-category**						< 0.001
T1	757 (37.5%)	192 (9.5%)	52 (10.5%)	8 (20.5%)	132 (9.0%)	
T2	804 (39.8%)	1136 (56.6%)	271 (54.4%)	24 (61.5%)	841 (57.2%)	
T3	368 (18.2%)	592 (29.5%)	137 (27.5%)	6 (15.4%)	449 (30.5%)	
T4	91 (4.5%)	88 (4.4%)	38 (7.6%)	1 (2.6%)	49 (3.3%)	
**N-category**						< 0.001
N0	1022 (50.6%)	596 (29.7%)	121 (24.3%)	13 (33.3%)	462 (31.4%)	
N1	677 (33.5%)	1144 (57.0%)	271 (54.4%)	20 (51.3%)	853 (58.0%)	
N2	237 (11.7%)	209 (10.4%)	75 (15.1%)	4 (10.3%)	130 (8.8%)	
N3	84 (4.2%)	59 (2.9%)	31 (6.2%)	2 (5.1%)	26 (1.8%)	
**Grade**						< 0.001
Well/Moderately differentiated	907 (44.9%)	728 (36.3%)	137 (27.5%)	13 (33.3%)	578 (39.3%)	
Poorly differentiated/Undifferentiated	1053 (52.1%)	1114 (55.5%)	336 (67.5%)	21 (53.9%)	757 (51.5%)	
Others	60 (3.0%)	166 (8.2%)	25 (5.0%)	5 (12.8%)	136 (9.2%)	
**No. of LN examination**						0.712
Mean	17.5	17.3	21.0	12.2	16.3	
Median (range)	15 (1–79)	15 (1–81)	18 (1–81)	12 (1–31)	15 (1–68)

The mean number of dissected LNs was 17.5 among patients without neoadjuvant therapy and 17.3 among patients with neoadjuvant therapy (*P* > 0.05). In detail, patients with neoadjuvant chemotherapy had the highest mean number of dissected LNs (21.0, *P* < 0.001), while those with neoadjuvant radiotherapy had the lowest mean number of dissected LNs (12.2, *P* = 0.003). The mean LN number of patients with neoadjuvant chemoradiotherapy was 16.3, which was still significantly lower than 17.5 for patients without neoadjuvant therapy (*P* = 0.001).

### Optimal number of LN examination

**Fig. 2:**
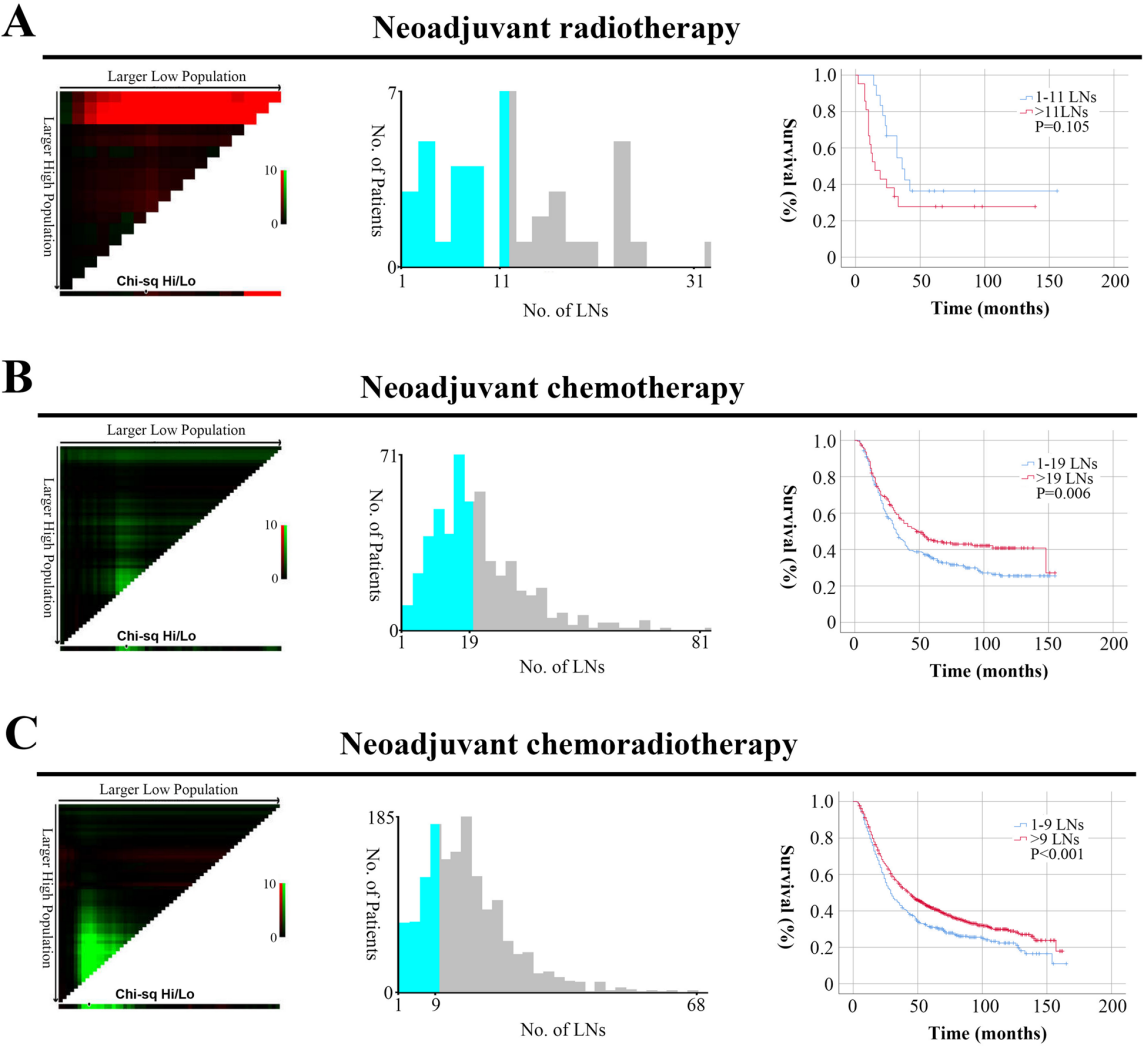
The optimal number of LN examination calculated using X-tile. **a** patients with neoadjuvant radiotherapy; **b** patients with neoadjuvant chemotherapy; **c** patients with neoadjuvant chemoradiotherapy

We determined the optimal cutoff value for the number of dissected LNs using X-tile analysis. The optimal cutoff value was 11 for patients with neoadjuvant radiotherapy. However, the survival of patients with 1–11 LNs and those with > 11 LNs did not differ (Fig. 2A, *P *> 0.05). This negative result might be related to the small sample size (39 cases). For patients with neoadjuvant chemotherapy, the optimal cutoff value was 19. Patients with > 19 LNs had a better prognosis than those with 1–19 LNs (Fig. 2B, *P* < 0.05). For patients with neoadjuvant chemoradiotherapy, the optimal cutoff value was 9. Patients with > 9 LNs had a better prognosis than those with 1–9 LNs (Fig. 2C, *P* < 0.05). Therefore, we divided the entire cohort into three groups according to the optimal number of LN examination: 1–9, 10–19, and > 19 LNs.

### COX regression analyses

Furthermore, we performed univariate and multivariate COX regression analyses for patients with neoadjuvant chemotherapy and chemoradiotherapy. We did not include patients with neoadjuvant radiotherapy due to the low sample size. The univariate analysis demonstrated that grade (well/moderately differentiated vs. poorly differentiated/undifferentiated), T-category, and N-category were prognostic factors for patients with neoadjuvant chemotherapy (Table 2). The multivariate analysis demonstrated that the T-category (T1 vs. T3), N-category, and the number of LN examination (1–9 vs. > 19) were independent prognostic factors for them (Table 2). The univariate analysis demonstrated that age, sex, grade (well/moderately differentiated vs. poorly differentiated/undifferentiated), marital status, T-category, N-category, and the number of LN examination were prognostic factors for patients with neoadjuvant chemoradiotherapy (Table 3). Further multivariate analysis demonstrated that age, sex, grade (well/moderately differentiated vs. poorly differentiated/undifferentiated), marital status, N-category, and the number of LN examination were independent prognostic factors for them (Table 3).

**Table 2:** Univariate and multivariate analyses for OS in EGC patients with neoadjuvant chemotherapy

**Characteristics**	**Univariate Analysis**			**Multivariate Analysis**		
	**HR**^**a**^	**95%CI**^**b**^	***P*** **-value**	**HR**^**a**^	**95%CI**^**b**^	***P*** **-value**
**Age**						
≤ 60	Reference			Reference		
> 60	1.033	0.826–1.291	0.778	1.210	0.959–1.528	0.109
**Year of diagnosis**						
2006–2011	Reference			Reference		
2012–2017	0.900	0.717–1.130	0.363	0.871	0.687–1.103	0.250
**Sex**						
Male	Reference			Reference		
Female	0.930	0.690–1.252	0.631	0.922	0.676–1.256	0.605
**Race**						
White	Reference			Reference		
Black	0.775	0.461–1.305	0.338	0.635	0.369–1.091	0.100
Others	0.848	0.578–1.244	0.400	0.827	0.555–1.232	0.351
**Grade**						
Well/moderately differentiated	Reference			Reference		
Poorly differentiated/undifferentiated	1.416	1.094–1.831	0.008	1.298	0.986–1.709	0.063
Others	1.102	0.634–1.914	0.731	1.145	0.652–2.012	0.637
**Histology**						
Adenocarcinomas	Reference			Reference		
Others	1.270	0.972–1.659	0.080	1.107	0.831–1.473	0.487
**Marital status**						
Married	Reference			Reference		
Others	1.100	0.864–1.401	0.439	1.266	0.983–1.630	0.068
**T-category**						
T1	Reference			Reference		
T2	1.659	1.079–2.550	0.021	1.484	0.946–2.328	0.086
T3	2.674	1.713–4.173	< 0.001	2.019	1.255–3.246	0.004
T4	2.292	1.299–4.043	0.004	1.815	0.993–3.317	0.053
**N-category**						
N0	Reference			Reference		
N1	1.705	1.262–2.305	0.001	1.488	1.080–2.049	0.015
N2	2.649	1.828–3.838	< 0.001	2.377	1.593–3.547	< 0.001
N3	4.133	2.565–6.660	< 0.001	4.306	2.562–7.237	< 0.001
**No. of LN examination**						
1–9	Reference			Reference		
10–19	1.248	0.891–1.747	0.198	0.933	0.655–1.329	0.701
> 19	0.864	0.616–1.212	0.397	0.593	0.412–0.854	0.005

^a^*HR* hazard ratio

^b^*CI* confidence interval

**Table 3:** Univariate and multivariate analyses for OS in EGC patients with neoadjuvant chemoradiotherapy

**Characteristics**	**Univariate Analysis**			**Multivariate Analysis**		
	**HR**^**a**^	**95%CI**^**b**^	***P*** **-value**	**HR**^**a**^	**95%CI**^**b**^	***P*** **-value**
**Age**						
≤ 60	Reference			Reference		
> 60	1.171	1.030–1.332	0.016	1.197	1.051–1.363	0.007
**Year of diagnosis**						
2006–2011	Reference			Reference		
2012–2017	0.894	0.786–1.017	0.088	0.878	0.769–1.001	0.051
**Sex**						
Male	Reference			Reference		
Female	0.766	0.636–0.923	0.005	0.768	0.636–0.928	0.006
**Race**						
White	Reference			Reference		
Black	0.722	0.485–1.074	0.108	0.768	0.513–1.150	0.200
Others	0.794	0.587–1.075	0.136	0.853	0.629–1.158	0.309
**Grade**						
Well/moderately differentiated	Reference			Reference		
Poorly differentiated/undifferentiated	1.360	1.190–1.553	< 0.001	1.331	1.158–1.530	< 0.001
Others	0.863	0.674–1.105	0.244	0.872	0.680–1.117	0.278
**Histology**						
Adenocarcinomas	Reference			Reference		
Others	1.179	0.996–1.395	0.056	1.017	0.853–1.212	0.854
**Marital status**						
Married	Reference			Reference		
Others	1.212	1.058–1.390	0.006	1.296	1.129–1.488	< 0.001
**T-category**						
T1	Reference			Reference		
T2	1.418	1.111–1.809	0.005	1.165	0.906–1.499	0.233
T3	1.347	1.042–1.741	0.023	1.088	0.834–1.418	0.534
T4	1.977	1.335–2.926	0.001	1.451	0.968–2.175	0.072
**N-category**						
N0	Reference			Reference		
N1	1.538	1.329–1.779	< 0.001	1.530	1.315–1.780	< 0.001
N2	1.853	1.462–2.349	< 0.001	1.951	1.527–2.494	< 0.001
N3	2.994	1.951–4.593	< 0.001	2.821	1.824–4.363	< 0.001
**No. of LN examination**						
1–9	Reference			Reference		
10–19	0.786	0.676–0.913	0.002	0.758	0.651–0.882	< 0.001
> 19	0.736	0.622–0.871	< 0.001	0.672	0.566–0.797	< 0.001

^a^*HR* hazard ratio

^b^*CI* confidence interval

## Discussion

EGC is a lethal disease with an increasing incidence and a poor prognosis [18]. Considering the location and histological characteristics, EGC does not completely resemble esophageal or gastric cancer, raising debates on surgical margins [19]. Therefore, distinguishing EGC as a specific type of malignant tumor of the digestive tract has become crucial to provide insights into its clinical properties. LN metastases occur in more than 20% of T1-category patients and are considered a negative prognostic factor [20, 21]. Therefore, lymphadenectomy is a critical part of surgical treatment.

Herein, we evaluated the impact of neoadjuvant therapy on the number of dissected LNs in EGC patients using the SEER database. We found that neoadjuvant radiotherapy and chemoradiotherapy decreased the number of dissected LNs in EGC patients, while neoadjuvant chemotherapy increased it. Our results were consistent with previous studies regarding esophageal cancer in which radiotherapy/chemoradiotherapy would cause a decrease in LN number [22–24]. These results might be related to tumor and nodal down-staging, which also has been proved in other cancers, including rectal and non-small cell lung cancer [25, 26]. However, the impact of neoadjuvant chemotherapy alone on the number of dissected LNs in EGC or esophageal cancer has not been reported. In breast cancer, Boughey et al. [27] found that neoadjuvant chemotherapy increased the number of axillary LN dissection (21.9 vs. 20.2) although the difference did not achieve statistical significance. Additionally, other studies have shown that neoadjuvant chemotherapy is associated with a lower axillary LN count in breast cancer [28, 29]. These inconsistent results indicated that the number of LN dissection might not be strongly associated with chemotherapy. One possible explanation is that patients with neoadjuvant chemotherapy might have more severe nodal diseases. Based on our data from Table 1, N2 and N3 categories accounted for 21.3% of neoadjuvant chemotherapy while 10.6% in patients with neoadjuvant chemoradiotherapy. Moreover, the optimal cutoff value of LN dissection was 19 for patients with neoadjuvant chemotherapy, much higher than neoadjuvant chemoradiotherapy. These findings indicated that patients with neoadjuvant chemotherapy alone might undergo appropriate or even radical procedures to obtain more LNs.

Numerous data, including National Comprehensive Cancer Network (NCCN) guidelines, have indicated that at least 15 LNs should be dissected for patients without neoadjuvant therapy to guarantee accurate staging and survival benefits [23, 30–32]. However, the optimal number of LN dissection for patients with neoadjuvant chemoradiotherapy remains controversial. Samson et al. [23] found that removing 10–15 LNs brought additional survival benefits for esophageal cancer patients with neoadjuvant chemoradiotherapy. Another institutional analysis showed that esophageal cancer patients with > 7 LN examination had a better prognosis than those with 1–7 LNs [33]. Unfortunately, no relevant literature about the impact of neoadjuvant chemoradiotherapy on the LN count has been reported in EGC patients. In the present study, we recommended that at least 10 LNs be removed since the greatest survival difference was reached at the cut-off point (1–9 LNs vs. > 9 LNs). Different from patients with neoadjuvant chemotherapy alone, surgeons should avoid an extremely radical operation as much as possible for patients with neoadjuvant chemoradiotherapy. The removal of more LNs might increase the risk of complications without adding survival benefits.

However, our current study also has some limitations. First, this was a retrospective study with inherent limitations such as selection bias. Hence, a large prospective clinical trial is needed to validate our conclusions. Second, detailed chemoradiotherapy information was missing, including the type and dose of radiotherapy and chemotherapy, delivery methods, and duration of treatment, which might have some impact on the conclusions. Third, the SEER database has no quality-of-life information, which can not be ignored while treating malignant patients.

## Conclusion

Neoadjuvant radiotherapy and chemoradiotherapy decreased the number of dissected LNs, while neoadjuvant chemotherapy increased it in EGC patients. Hence, at least 10 LNs should be dissected for neoadjuvant chemoradiotherapy and 20 for neoadjuvant chemotherapy, which could be applied in clinical practice.

## Data Availability

All data are derived from the SEER database produced by the National Cancer Institute (https://seer.cancer.gov/) Program. Detailed steps: (1) apply for the use of SEER; (2) following approval by SEER, relevant account and password will be sent to you via E-mail; (3) visit the database using the SEER*Stat software and download the patient data.

## References

[1] Yamashita K, Sakuramoto S, Nemoto M, Shibata T, Mieno H, Katada N (2011). Trend in gastric cancer: 35 years of surgical experience in Japan. World J Gastroenterol.

[2] Devesa SS, Blot WJ, Fraumeni JF (1998). Changing patterns in the incidence of esophageal and gastric carcinoma in the United States. Cancer.

[3] Anderegg MC, Lagarde SM, Jagadesham VP, Gisbertz SS, Immanuel A, Meijer SL (2016). Prognostic significance of the location of lymph node metastases in patients with adenocarcinoma of the distal esophagus or gastroesophageal junction. Ann Surg.

[4] Yamashita H, Katai H, Morita S, Saka M, Taniguchi H, Fukagawa T (2011). Optimal extent of lymph node dissection for Siewert type II esophagogastric junction carcinoma. Ann Surg.

[5] Fujitani K, Miyashiro I, Mikata S, Tamura S, Imamura H, Hara J (2013). Pattern of abdominal nodal spread and optimal abdominal lymphadenectomy for advanced Siewert type II adenocarcinoma of the cardia: results of a multicenter study. Gastric Cancer.

[6] Wang JB, Lin MQ, Li P, Xie JW, Lin JX, Lu J (2017). The prognostic relevance of parapyloric lymph node metastasis in Siewert type II/III adenocarcinoma of the esophagogastric junction. Eur J Surg Oncol.

[7] Peng J, Wang WP, Yuan Y, Hu Y, Wang Y, Chen LQ (2015). Optimal Extent of Lymph Node Dissection for Siewert Type II Esophagogastric Junction Adenocarcinoma. Ann Thorac Surg.

[8] Okholm C, Fjederholt KT, Mortensen FV, Svendsen LB, Achiam MP (2018). The optimal lymph node dissection in patients with adenocarcinoma of the esophagogastric junction. Surg Oncol.

[9] Bekkar S, Gronnier C, Messager M, Robb WB, Piessen G, Mariette C (2014). The impact of preoperative radiochemotherapy on survival in advanced esophagogastric junction signet ring cell adenocarcinoma. Ann Thorac Surg.

[10] Rice TW, Lerut TE, Orringer MB, Chen LQ, Hofstetter WL, Smithers BM (2016). Worldwide Esophageal Cancer Collaboration: neoadjuvant pathologic staging data. Dis Esophagus.

[11] Pericay C, Macias-Declara I, Arrazubi V, Vila L, Marin M (2019). Treatment in esophagogastric junction cancer: past, present and future. Cir Esp (Engl Ed).

[12] Kelly RJ, Ajani JA, Kuzdzal J, Zander T, Van Cutsem E, Piessen G (2021). Adjuvant nivolumab in resected esophageal or gastroesophageal junction cancer. N Engl J Med.

[13] Kang YK, Yook JH, Park YK, Lee JS, Kim YW, Kim JY (2021). PRODIGY: A Phase III Study of Neoadjuvant Docetaxel, Oxaliplatin, and S-1 Plus Surgery and Adjuvant S-1 Versus Surgery and Adjuvant S-1 for Resectable Advanced Gastric Cancer. J Clin Oncol.

[14] Noordman BJ, Verdam MGE, Lagarde SM, Hulshof M, van Hagen P, van Berge Henegouwen MI (2018). Effect of Neoadjuvant Chemoradiotherapy on Health-Related Quality of Life in Esophageal or Junctional Cancer: Results From the Randomized CROSS Trial. J Clin Oncol.

[15] Tang Z, Wang Y, Liu D, Wang X, Xu C, Yu Y (2022). The Neo-PLANET phase II trial of neoadjuvant camrelizumab plus concurrent chemoradiotherapy in locally advanced adenocarcinoma of stomach or gastroesophageal junction. Nat Commun.

[16] Issaka A, Ermerak NO, Bilgi Z, Kara VH, Celikel CA, Batirel HF (2015). Preoperative chemoradiation therapy decreases the number of lymph nodes resected during esophagectomy. World J Surg.

[17] Wu SG, Zhang ZQ, Liu WM, He ZY, Li FY, Lin HX (2016). Impact of the number of resected lymph nodes on survival after preoperative radiotherapy for esophageal cancer. Oncotarget.

[18] Lagergren J, Lagergren P (2013). Recent developments in esophageal adenocarcinoma. CA Cancer J Clin.

[19] Gertler R, Stein HJ, Schuster T, Rondak IC, Hofler H, Feith M (2014). Prevalence and topography of lymph node metastases in early esophageal and gastric cancer. Ann Surg.

[20] Zhang M, Li Z, Ma Y, Zhu G, Zhang H, Xue Y (2012). Prognostic predictors of patients with carcinoma of the gastric cardia. Hepatogastroenterology.

[21] Pedrazzani C, de Manzoni G, Marrelli D, Giacopuzzi S, Corso G, Minicozzi AM (2007). Lymph node involvement in advanced gastroesophageal junction adenocarcinoma. J Thorac Cardiovasc Surg.

[22] Mariette C, Piessen G, Briez N, Triboulet JP (2008). The number of metastatic lymph nodes and the ratio between metastatic and examined lymph nodes are independent prognostic factors in esophageal cancer regardless of neoadjuvant chemoradiation or lymphadenectomy extent. Ann Surg.

[23] Samson P, Puri V, Broderick S, Patterson GA, Meyers B, Crabtree T (2017). Extent of lymphadenectomy is associated with improved overall survival after esophagectomy with or without induction therapy. Ann Thorac Surg.

[24] Kauppila JH, Wahlin K, Lagergren P, Lagergren J (2018). Neoadjuvant therapy in relation to lymphadenectomy and resection margins during surgery for oesophageal cancer. Sci Rep.

[25] van Eeghen EE, Bakker SD, Fransen G, Flens MJ, Loffeld R (2017). Tumor stage in patients operated for rectal cancer: a comparison of the pre-operative MR and the resection specimen, with specific attention to the effect of neo-adjuvant radiotherapy. J Gastrointest Oncol.

[26] Yalman D (2015). Neoadjuvant radiotherapy/chemoradiotherapy in locally advanced non-small cell lung cancer. Balkan Med J.

[27] Boughey JC, Donohue JH, Jakub JW, Lohse CM, Degnim AC (2010). Number of lymph nodes identified at axillary dissection: effect of neoadjuvant chemotherapy and other factors. Cancer.

[28] Belanger J, Soucy G, Sideris L, Leblanc G, Drolet P, Mitchell A (2008). Neoadjuvant chemotherapy in invasive breast cancer results in a lower axillary lymph node count. J Am Coll Surg.

[29] Neuman H, Carey LA, Ollila DW, Livasy C, Calvo BF, Meyer AA (2006). Axillary lymph node count is lower after neoadjuvant chemotherapy. Am J Surg.

[30] Schwarz RE, Smith DD (2007). Clinical impact of lymphadenectomy extent in resectable esophageal cancer. J Gastrointest Surg.

[31] Samson P, Puri V, Robinson C, Lockhart C, Carpenter D, Broderick S (2016). Clinical T2N0 Esophageal Cancer: Identifying Pretreatment Characteristics Associated With Pathologic Upstaging and the Potential Role for Induction Therapy. Ann Thorac Surg.

[32] Bollschweiler E, Baldus SE, Schroder W, Schneider PM, Holscher AH (2006). Staging of esophageal carcinoma: length of tumor and number of involved regional lymph nodes. Are these independent prognostic factors?. J Surg Oncol.

[33] Hanna JM, Erhunmwunsee L, Berry M, D'Amico T, Onaitis M (2015). The prognostic importance of the number of dissected lymph nodes after induction chemoradiotherapy for esophageal cancer. Ann Thorac Surg.

